# Silica nanoparticles produced by explosive flash vaporization during earthquakes

**DOI:** 10.1038/s41598-019-46320-7

**Published:** 2019-07-05

**Authors:** Takashi Amagai, Atsushi Okamoto, Takamasa Niibe, Nobuo Hirano, Kenichi Motomiya, Noriyoshi Tsuchiya

**Affiliations:** 1 Graduate School of Environmental Studies, Tohoku University, 6-6-20, Aramaki-Aza-Aoba, Aoba-ku, Sendai, Miyagi 980-8579 Japan; 2 Japan Oil, Gas and Metals National Corporation, 10-1, Toranomon 2-chome, Minato-ku, Tokyo 105-0001 Japan

**Keywords:** Geochemistry, Mineralogy, Petrology

## Abstract

Hydrothermal activity in the crust results in the precipitation of large volumes of silica and often involves the formation of ore deposits, the shaping of geothermal systems, and recurring earthquakes. Pore fluid pressures fluctuate between lithostatic and hydrostatic, depending on seismic activity, and some models suggest the possibility of flash vaporization, given that fluid pressures can drop to the level of vapour at fault jogs during seismic slip. The phase changes of water could create extremely high supersaturations of silica, but the mechanisms of quartz vein formation under such extreme conditions remain unclear. Here we describe flash experiments conducted with silica-saturated solutions under conditions ranging from subcritical to supercritical. We found that amorphous silica is produced instantaneously as spherical nano- to micron-scale particles via nucleation and aggregation during the evaporation of water droplets. The nanoparticles are transformed to microcrystalline quartz very rapidly by dissolution and precipitation in hydrothermal solutions, with this process requiring less than one day under supercritical conditions because of the huge surface areas involved. We suggest that such short-lived silica nanoparticles have significant impacts on the dynamic changes in mechanical behaviour and hydrology of hydrothermal systems in volcanic areas.

## Introduction

Fluid flow along fractures and faults plays an essential role in the transport of energy and the redistribution of elements within the upper crust, and the chemical interactions between hydrothermal fluids and rocks influence the mechanical and hydrological properties of the crust, as is evident in the formation of hydrothermal ore deposits^1,2^, the shaping and maintenance of geothermal systems^3–5^, and recurring earthquakes^6^.

Quartz occurs ubiquitously as a fracture-filling mineral, as silica is the most abundant component of the crust, and the solubility of quartz in water is strongly dependent on temperature and pressure^7,8^. There are several mechanisms of quartz vein formation, including the upflow of deep-seated fluids along fracture networks and diffusion from the host rocks without significant fluid advection^9,10^. Mineralogical and fluid inclusion studies of epithermal or mesothermal gold–quartz veins have revealed seismically induced fluctuations in fluid pressure between lithostatic and hydrostatic conditions in the crust, thereby causing the boiling of hydrothermal fluids and silica precipitation^11,12^. Weatherley and Henley^13^ proposed a model in which gold–quartz veins form by the flash vaporization of fluids at the very instant of earthquake rupturing. In their model, the fluids could be decompressed to the level of vapour at a fault jog. They speculated that gold and silica particles could be co-precipitated by the flash vaporization. However, this interesting hypothesis has yet to be proven.

Despite quartz being thermodynamically stable at the Earth’s surface and in the crust, it is known that metastable silica polymorphs also occur commonly at the surface or in subsurface deposits at temperatures of <100 °C in association with hot springs and seafloor hydrothermal vents^14^. During diagenesis, amorphous silica (opal-A) in a deposit may transform into more stable phases, with cristobalite (opal-C/CT) and chalcedony changing to quartz over a period of >1000 years^14^. The question arises as to whether quartz veins may form via amorphous silica, even under hydrothermal conditions in the crust at higher temperatures of T > 200 °C. Some quartz veins are filled with crystals grown from the vein walls, but other types show mosaic (blocky) textures or textures composed of microcrystalline quartz^10^. The microcrystalline type is thought to have developed through various mechanisms, including plastic deformation of quartz grains and precipitation of amorphous silica (as a precursor) from highly supersaturated solutions^15–17^. Metastable silica phases usually contain water, which is easily lost during deformation and transformation into more stable phases. However, the metastable silica phases themselves are not preserved in most quartz veins.

Experimental studies of silica precipitation remain limited. Flow-through experiments^18–20^ have revealed that various types of silica precipitation occur via a range of mechanisms under supercritical conditions including epitaxial quartz overgrowth, formation of metastable silica minerals, and 3-dimensional homogeneous (or heterogeneous) nucleation of quartz in fluids, depending on temperature and solution chemistry. In the field of engineering, silica nanoparticles have been synthesized by the rapid expansion of silica-dissolved solutions^21^ or the evaporation of silica gel dispersed droplets^22^, but their geological significance has not been considered.

The aim of this study was to understand the mechanism of silica precipitation induced by flash vaporization, and to evaluate whether the formation of amorphous silica is a key step in the formation of quartz veins in the crust. We conducted novel experiments on quartz formation comprising two steps: flashing experiments under subcritical or supercritical fluid to air pressures with granite-dissolved aqueous solutions, and batch-type experiments to investigate the transformation of the flashing products under hydrothermal conditions.

## Results

### Silica particles produced by flashing experiments

**Figure 1:**
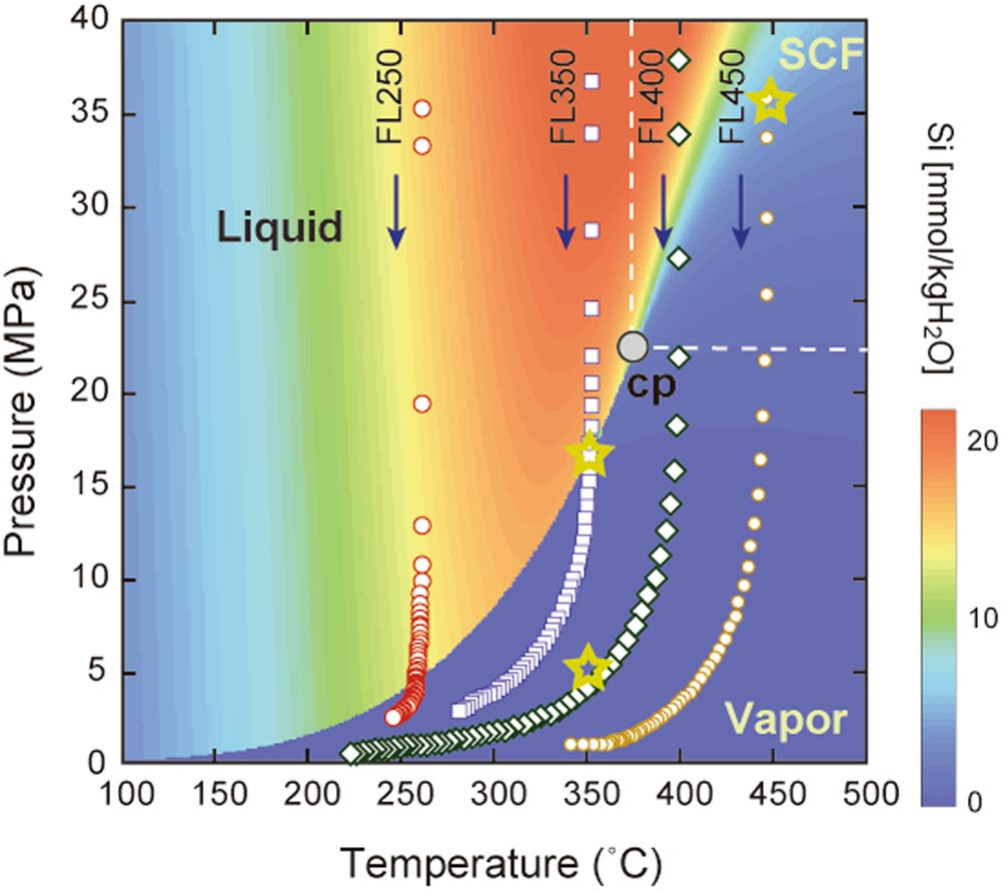
Quartz solubility in water on a phase diagram for water, and P–T conditions of the flashing and batch experiments. The solubility of quartz in water is from Manning^7^. The P–T paths were for 5 s after flashing at 261 °C and 35.2 MPa (run FL250), 353 °C and 36.7 MPa (FL350), 400 °C and 37.8 MPa (FL400), and 450 °C and 35.8 MPa (FL450). Each P–T path was recorded at 0.1 s intervals. Yellow open stars indicate P–T conditions of the batch experiments for the transformation of flashing products. SCF = supercritical fluid; cp = critical point.

Four flashing experiments were conducted under T–P conditions of 261 °C and 35.2 MPa (run FL250), 353 °C and 36.7 MPa (FL350), 400 °C and 37.8 MPa (FL400), and 450 °C and 35.8 MPa (FL450). All runs showed nearly isothermal decompression paths within the first 1.5 seconds, with or without a phase transition of H_2_O (Fig. 1). In runs FL250 and FL350, the P–T paths crossed the liquid–vapour boundary of water. In runs FL400 and FL450, the flashing did not cause a phase transition of water, but the water density decreased gradually from a supercritical fluid to vapour. Within 5 seconds after the isothermal decompression stage, the temperature within the autoclave decreased slightly with decreasing pressure due to the latent heat induced by the evaporation of liquid water in the inlet capillary tube.

**Figure 2:**
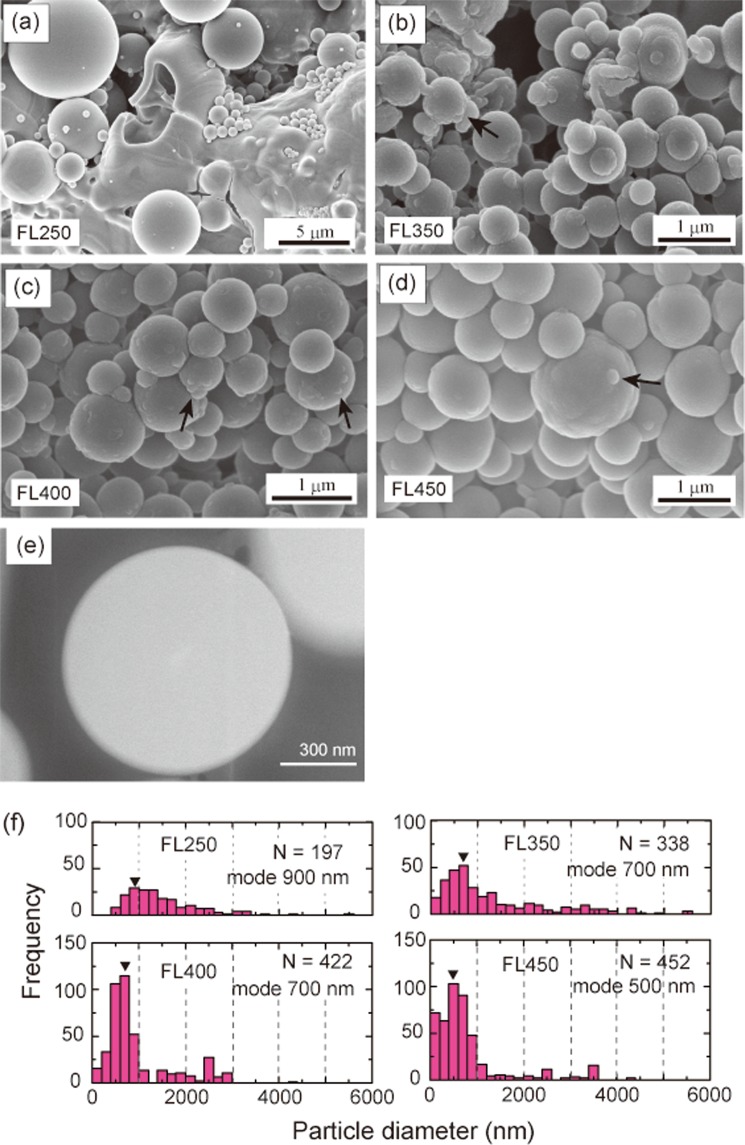
Surface morphology and size of precipitates produced by the flashing experiments. (**a**–**d**) SEM images of the surface morphology of the products in the flashing experiments for runs (**a**) FL250, (**b**) FL350, (**c**) FL400, and (**d**) FL450. Black arrows indicate the cauliflower-like roughness produced by aggregation. (**e**) Dark-field image of silica particles produced by the flashing experiment at 400 °C (same conditions as in run FL400). (**f**) Histograms showing the size distribution of silica particles for the four flashing experiments, measured from SEM images.

Silica precipitates were caught by the alumina filter that was placed at the outlet of the autoclave (Fig. S1). In all runs, the spherical silica particles collided and became stacked on the surface of the alumina filter (Fig. 2a–e). Raman spectra of the silica precipitates display a broad peak at 300–550 cm^−1^, indicating that the particles comprise amorphous silica^23^ (Fig. S2). The diameters of the silica particles range from ~100 to ~6000 nm (Fig. 2f). The particle size distributions observed in the SEM images are not normal distributions, but are skewed to smaller sizes. The mode of the particle size was 900 nm in run FL250, 700 nm in FL350 and FL400, and 500 nm in FL450. Most particles had relatively smooth spherical surfaces, but some displayed a cauliflower-like roughness with a wavelength of 100–200 nm (Fig. 2c,d). Highly angular scanning transmission electron microscope (STEM; Hitachi HD-2700A) dark-field (or Z-contrast) images indicate that the spherical silica particles have uniform internal structures without nano-scale pores or evidence of characteristic growth patterns (Fig. 2e).

**Figure 3:**
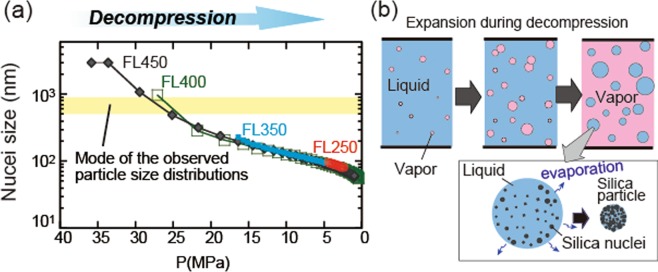
Conditions and model of formation of silica particles in the flashing experiments. (**a**) Predicted nucleus size during the flashing experiments along the P–T paths of the flashing experiments based on the classical nucleation theory^24^. Yellow band indicates the mode of the silica particle size distributions in Fig. 2e. (**b**) Schematic illustration of the formation of silica particles during flashing.

In classical nucleation theory, the critical radius of the cluster, *r*_c_, for homogeneous nucleation is as follows^24^:

1 $${r}_{c}=\frac{2\sigma \nu }{{\rm{RT}}ln{\rm{\Omega }}}$$

where σ, *v*, and Ω indicate the interfacial energy between the mineral and water, the molar volume, and the saturation ratio of the mineral, respectively. *R* is the universal gas constant, and *T* is temperature (K). In our experiments, amorphous silica nucleated instead of quartz in all runs, because of the lower interfacial energy of amorphous silica with water (65 mJ m^−2^) compared with that of quartz (350 mJ m^−2^; ref.^24^). Equation 1 also indicates that the nucleus size decreases with increasing temperature and with an increase in the saturation ratio. Equation 1 predicts that an extremely high supersaturation at the phase transition of H_2_O results in the formation of fine nuclei (100–200 nm in run FL250 and 30–200 nm in FL350), whereas during flashing from supercritical fluids (runs FL400 and FL450) the continuous nucleation of amorphous silica is expected to occur over a wide range of P–T conditions during gradual changes in water density, resulting in a wide range of nucleus sizes (30–1000 nm diameter; Fig. 3a). However, the observed particle size distributions of silica particles (Fig. 2f) cannot be explained solely by homogeneous nucleation theory. In particular, the large particles in run FL250 (mode 700–900 nm, maximum ~6000 nm) deviate from the predicted size (100–200 nm). The large range of particle sizes in run FL250 (Fig. 2a) suggests that nucleation occurred in at least two separate events with supersaturation having passed through two maxima during pressure release, although the detailed mechanism is unclear.

The cauliflower-like surface morphology of the silica particles (arrows in Fig. 2b–d) suggests that the aggregation of fine silica particles occurred during flashing. Iskandar *et al*.^22^ created silica particles by spray-drying a nanoparticle sol, and showed that the sizes and shapes of the silica particles were controlled by the sizes of water droplets and the concentrations of silica particles. In our experiments, the silica existed as a monomer in the input solution, and during flashing liquid water was fragmented into numerous droplets. In each droplet, many nuclei of amorphous silica were produced, and they would have aggregated to form a particle with a size that exceeded the size predicted by nucleation theory (Fig. 3b).

### Transformation of silica particles by dissolution and precipitation processes

**Figure 4:**
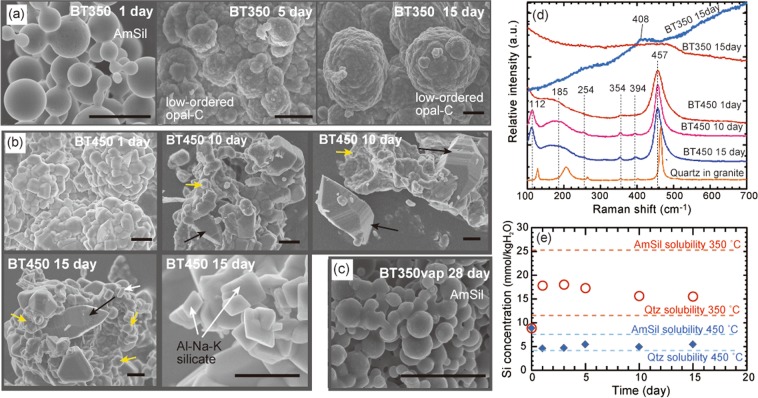
Results of batch experiments on silica transformation. (**a**–**c**) Surface morphology of products: (**a**) after days 1, 5 and 15 in run BT350; (**b**) after days 1, 10 and 15 in run BT450. Yellow and black arrows indicated poorly crystalline rounded silica phases and well-faceted quartz crystals, respectively. White arrows indicate platy Al-Na-K silicate. (**c**) Run BT350vap (vapour condition of water; 350 °C; 5 MPa). Amorphous silica remains even after day 28. AmSil = amorphous silica; Qtz = quartz. Scale bar indicates 2 μm. (**d**) Raman spectra of products of run BT350 at day 15, and of run BT450 at days 1, 10 and 15. Spectra of quartz grains in Iidate granite used for preparing the input solution are shown for comparison. The spectra of synthetic quartz shifted to lower wavenumbers than the quartz in granite. (**e**) Si concentration (mmol kg_H2O_^−1^) in runs BT350 and BT450. Broken lines of the same colour indicate the solubility of quartz (lower value) and amorphous silica (higher value) under particular P–T conditions.

We conducted batch experiments at 350 °C and 16 MPa (vapour-saturated pressure), and 450 °C and 36 MPa (extension of the vapour-saturated curve under supercritical conditions) to investigate the transformation of amorphous silica particles produced by flashing (Fig. 1). The silica mineral changed in different ways during runs BT350 (Fig. 4a) and BT450 (Fig. 4b). For comparative purposes, we also conducted a run under vapour conditions of 350 °C and 5 MPa (run BT350vap; Fig. 4c). Resulting Raman spectra are shown in Fig. 4d.

In run BT350, the amorphous silica occurred as spherical particles on day 1, with adjacent particles being connected to form necking structures (Fig. 4a). Semi-quantitative analyses by energy-dispersive X-ray spectrometry (EDXS) revealed that these particles contained 95–99 wt.% SiO_2_ with minor amounts of Al_2_O_3_ (<1 wt.%), Na_2_O (1.0–1.5 wt.%) and K_2_O (<1 wt.%) (Table S2). By day 5, surfaces of the silica precipitates had become rough and formed larger stacked aggregates of hemispherical particles (Fig. 4a). By day 15, these aggregates had sizes of up to 5–10 μm, with most displaying a broad amorphous-silica Raman peak, and some a weak peak at ~408 cm^−1^. The morphology and Raman spectra (Fig. 4a,d) suggest that amorphous silica was transformed into low-ordered opal-C^18,25^. The chemical composition of the opal-C was similar to that of the spherical amorphous silica (Table S1).

In the supercritical fluids (run BT450), the silica transformation occurred more rapidly. Even on day 1, hemispherical polygonal quartz aggregates (5–10 μm in size) were commonly present, with individual quartz crystals of 1–2 μm in size (Fig. 4b). These polygonal crystals displayed faceted surfaces with rounded edges. Some of the poorly-crystallized rounded silica phase remained even at day 15 (Fig. 4b), although by 10–15 days some quartz crystals had grown to 10–20 μm along their *c-*axes. These crystals developed crystal forms typical of quartz (Fig. 4b), with some being double-terminated. These well-faceted quartz crystals comprised almost 100 wt.% SiO_2_, while amorphous-like parts contained 1–2 wt.% Al_2_O_3_, Na_2_O and K_2_O (Table S2). Raman spectra display a main peak at ~457 cm^−1^, with minor peaks at ~112, ~185, 254, 354, and 394 cm^−1^ (Fig. 4d). Spectral peaks of synthetic quartz (Fig. 4d) occur at slightly smaller wavelengths to those of typical quartz crystals^25^ (464, 128, 206, 256, 355, and 394 cm^−1^). The peak at ~185 cm^−1^ is broad (Fig. 4d), probably due to the effects of low-crystalline components or impurities. Unidentified platy silicate minerals were also observed and contained Al_2_O_3_, Na_2_O and K_2_O (Fig. 4b; Table S2).

In the experiment under vapour conditions (run BT350vap), the spherical amorphous silica particles remained even after day 28 (Fig. 4c), although some were partly connected. This indicates that dissolution and precipitation processes in the solution played essential roles in the transformation of silica.

The Si concentration of the initial solution (*m*_Si_ = 9.19 mmol kg_H2O_^−1^) was lower than the solubility of quartz under the conditions of run BT350 (*m*_Si,eq,Qtz_ = 11.48 mmol kg_H2O_^−1^), but higher than the solubility of quartz in run BT450 (*m*_Si,eq,Qtz_ = 4.04 mmol kg_H2O_^−1^) (Fig. 4e). In run BT350, the Si concentration increased to its maximum (18.01 mmol kg_H2O_^−1^) by day 3, then decreased gradually to 15.51 mmol kg_H2O_^−1^ (Fig. 4e). In run BT450, the Si concentration decreased to 4.61 mmol kg_H2O_^−1^ by day 1, and was almost constant thereafter (4.6–5.4 mmol kg_H2O_^−1^) (Fig. 4e). The pH at room temperature did not change significantly during most experiments (from 6.8 before, to 6.7–7.2 after) (Table S1). Na concentrations were almost constant or decreased slightly (0.26–0.32 mmol kg_H2O_^−1^ in run BT350; 0.22–0.42 mmol kg_H2O_^−1^ in run BT450). Al and K concentrations (*m*_Al_ = 0.22 mmol kg_H2O_^−1^; *m*_K_ = 0.15 mmol kg_H2O_^−1^ in the initial solution) decreased, with the decreases being more pronounced in run BT450 (*m*_Al_ ≤ 0.01 mmol kg_H2O_^−1^; *m*_K_ = 0.07–0.08 mmol kg_H2O_^−1^, final solution) than in run BT350 (*m*_Al_ = 0.07–0.12 mmol kg_H2O_^−1^; *m*_K_ = 0.03–0.06 mmol kg_H2O_^−1^, final), which is largely consistent with the formation of Al-Na-K silicates (Fig. 4b; Table S2). Fe concentrations increased slightly to 0.02–0.11 mmol kg_H2O_^−1^ (Table S1), indicating minor leaching from the autoclave.

The transformation from amorphous silica to cristobalite and quartz has been investigated experimentally using silica gel^26^ and natural silica sediments^27^, but transformation rates have not been determined precisely because solid products rather than solutions were analysed. The change in Si concentration in run BT350 is explained by the coupled dissolution of amorphous silica and precipitation of cristobalite (low-ordered opal C). The dissolution of silica occurred more rapidly than cristobalite precipitation for the first three days, with the Si concentration increasing to a level exceeding cristobalite solubility (intermediate between those of quartz and amorphous silica). Then, with the increase in volume and surface area of the cristobalite, its precipitation rate exceeded the dissolution rate of amorphous silica, causing Si solubility to decrease towards that of cristobalite (Fig. 4e). This is consistent with the stepwise decrease in Si concentrations observed in experiments^28^ and predicted by models^29,30^ involving precipitation from amorphous silica-saturated solutions.

Cristobalite was not observed at 450 °C, but nucleation of quartz occurred rapidly at that temperature. As a result, the coupled dissolution of amorphous silica and precipitation of quartz caused a decrease in Si concentration to near the level of quartz solubility (Fig. 4e). The preferential nucleation of quartz under supercritical conditions is consistent with results of flow-through experiments^5^. Impurities such as small amounts of Al, Na and K (Table S2), and water in amorphous silica phases might have facilitated the rapid nucleation of quartz^20^. The rate of precipitation of quartz as growth from substrate seed crystals, *R*_*SiO2*, *Qtz*_ (mol s^−1^), is simply expressed by the first order kinetic equation^31^ as follows:

2 $${R}_{SiO2,Qtz}={A}_{Qtz}{k}_{-}(Q-{K}_{Qtz{\rm{,}}{\rm{e}}{\rm{q}}})$$

where *k*_ is the precipitation rate constant (mol m^−2^ s^−1^), and *A*_*Qtz*_ is the reactive surface area of quartz (m^2^). *Q* and *K*_*Qtz*,eq_ respectively represent the activity product and its equilibrium constant for quartz. With using *k*_ = 2.2 × 10^−4^ mol m^−2^ s^−1^ at 450 °C^18^, when we consider the quartz precipitation from the 110 ml input solution (*m*_Si_ = 9.19 mmol kg_H2O_^−1^) on quartz surface with *A*_*Qtz*_ of 31 mm^2^ (the area of top of cylindrical alumina filter), it takes >50 days at 450 °C for the Si concentration to decrease to near the solubility of quartz (<5 mmol kg_H2O_^−1^). This means that quartz precipitation via amorphous silica, as observed in our two-step experiments, is much more rapid than has been estimated in models of quartz overgrowths on pre-existing crystals in vein walls, probably due to huge surface areas of amorphous silica and microcrystalline quartz (Figs 2, 4). Flashing and subsequent transformation might therefore play primary roles in quartz vein formation.

## Discussion

The vaporization of pore water requires drastic changes in the crustal environment, and such changes during seismic slip have been proposed to occur in two ways: by instantaneous decompression at a fault jog^13^, and by frictional heating on a fault surface^32^. Such vaporization causes a high degree of supersaturation of silica minerals in faults hosted by quartz-bearing crustal rocks. Our experiments have verified that flash vaporization produces a large number of nano- to micron-sized amorphous silica particles on the fault surface (Figs 2, 3). Recent observations of fault materials and the products of frictional experiments have suggested that silica gel is formed on the fault plane as the product of the amorphization of quartz during friction^17,33–36^, and that the amorphous silica acts as a lubricant that facilitates seismic slip^33^. We consider that amorphous silica is formed not only mechanically, but also by chemical processes during earthquake rupturing. Co-seismic slip also results in a continuous rise in fluid temperatures to >350 °C on fault surfaces, and this results in significant water–rock interaction^37^. Under such conditions, amorphous silica particles could change to aggregates of microcrystalline quartz within a few days, a process that may contribute to the recovery of fault strength.

**Figure 5:**
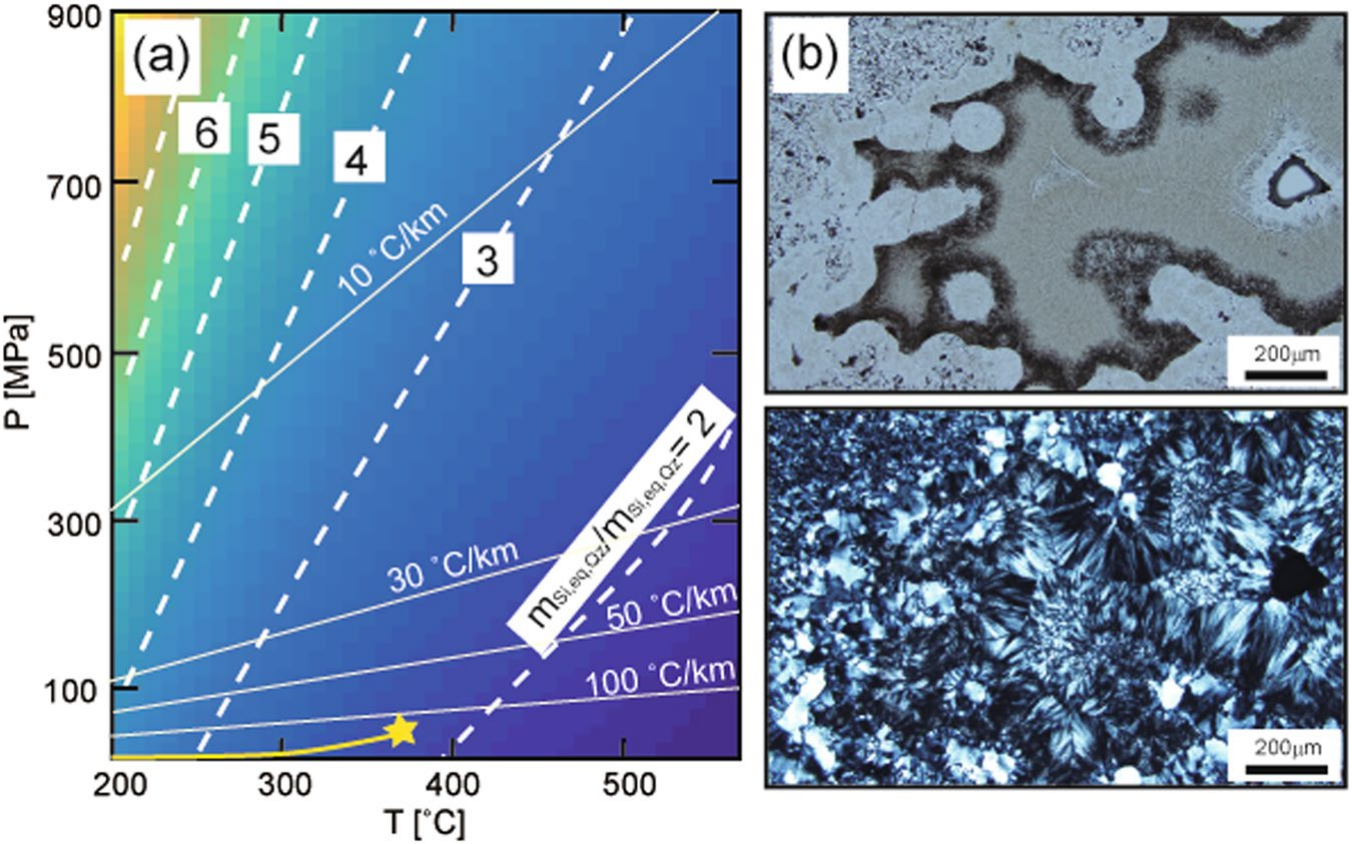
Solubility ratio of amorphous silica to quartz and photomicrograph of microcrystalline quartz within an epithermal deposit. (**a**) Contour diagram of the solubility ratio of amorphous silica (*m*_Si,eq,AS_) to quartz (*m*_Si,eq,Qz_) calculated following Karásek *et al*.^38^. Yellow curves and star indicate the vapour saturated curve and the critical point of water, respectively. White broken lines are contours of the solubility ratio, and white solid lines indicate the geothermal gradient. (**b**) Photomicrograph of an epithermal quartz vein: plane polarized light (top), crossed polarized light (bottom). The quartz vein is from the Hishikari Gold Mine (Japan) and is filled with microcrystalline quartz with spherical outlines of amorphous silica particles.

Since amorphous silica is not preserved in hydrothermal quartz veins, it remains unclear how common the formation of amorphous silica is within the crust. One simple constant regarding the formation of amorphous silica is that the fluid, which was originally in equilibrium with quartz, should exceed the solubility of amorphous silica at the time of vein formation. The solubility ratio of amorphous silica with respect to quartz (Fig. 5a; ref.^38^) increases with increasing pressure and decreasing temperature. Along a cold geothermal gradient (~10 °C km^−1^) such as a subduction zone interface, the solubility of amorphous silica is 3–5 times higher than that of quartz in temperature range of 200 °C–450 °C. Such a condition makes it difficult for amorphous silica to form even by the change from lithostatic to hydrostatic pressure^39^, except in extreme cases such as flash vaporization. In contrast, along a high geothermal gradient, such as in volcanic and geothermal areas where the gradient may exceed 100 °C km^−1^, the solubility of amorphous silica is only 1–2 times higher than that of quartz, which means that the formation of amorphous silica can be realized by subtle fluctuations in fluid pressure from lithostatic to hydrostatic, as well as boiling processes^12^. The scales of silica in the pipelines of geothermal power plants provide direct evidence for the formation of amorphous silica by flashing under such high temperatures. We suggest, therefore, that amorphous silica forms more commonly than expected, even within hot crust (T > 300 °C). Aggregates of nano-scale amorphous silica are short-lived, and are transformed to microcrystalline quartz with colloform or banded structures (Fig. 5b; refs^15,16^), as commonly observed in epithermal or mesothermal quartz veins.

One notable characteristic of silica nanoparticles produced by flashing is their high mobility with fluid flow, which is quite different from the silica that is precipitated as quartz overgrowth from vein walls. Recent seismological observations^2,40,41^ and continuous records of groundwater levels^42^ indicate that a redistribution of fluid pore pressures and fluid flow is induced by earthquakes, and that a dynamic oscillation of permeability is induced by distant earthquakes^42^. The permeability oscillation is often explained by the clogging and removal of clay mineral particles at pore throats on fault surfaces^43^. In the hydrothermal systems of volcanic areas, the flow of silica nanoparticles is easily self-organized as the result of oscillations in fluid pressure. We speculate that these silica nanoparticles are easily transported and can clog faults effectively, thus affecting the dynamic behaviour of the crust.

## Methods

### Input solutions

We used a single high-Si solution as the input solution for the flash and batch experiments. The solution was prepared by the dissolution of quartz (100 g) and granite sand (Iidate granite, Japan, 100 g) with a grain size of 1–2 mm in the flow-through apparatus. Distilled water entered the cylindrical autoclave at a constant flow rate of 0.7 mL min^−1^. The temperature was 370 °C and the fluid pressure was regulated by the back-pressure valve to be 41 MPa. The solution was cooled and stored in the plastic tank until each experiment. The concentrations of Si, Al, Na, K, Ca, Mg, and Fe in the input solutions and the solutions after the batch experiments were determined by inductively coupled plasma–atomic emission spectrometry (ICP–AES, Hitachi P-4000) at Tohoku University, Sendai, Japan. The solubility of quartz used in this study was calculated as a function of water density following Manning^7^. The solubility ratio of amorphous silica with respect to quartz is from Karásek *et al*.^38^. The pH of the input solution was 6.8 at room temperature (Table S1).

### Flash experiments

A cylindrical autoclave (volume 110 mL) was used for the flash experiments, and its inner wall and inner caps were made of a titanium alloy (Ti-6Al-4V) (Fig. S1a). The autoclave was placed vertically in the furnace, connected with a syringe pump on the inlet side, and connected to 6 mm internal diameter stainless steel tubes (SUS316) on the flashing side. The thermocouple was set at the centre of the autoclave and fluid pressure was measured at the inlet. To catch the silica particles, we installed a cylindrical alumina filter (Fuji Chemical, FA220, diameter 6.3 mm, height 5.0 mm, average pore diameter 60 μm) within the cap of the autoclave on the flashing side (Fig. S1a). Before flashing, the autoclave was filled with the input solution by the syringe pump at room temperature. The solution was then pressurized to ~36 MPa by controlling the back-pressure valve of the capillary line, and then heated to the target temperature. When the temperature reached the target temperature, the stop valve was opened. The fluid pressure instantaneously dropped to the air pressure, and the silica particles were caught by the alumina filter. We conducted a series of flashing experiments at temperatures of 261 °C at 35.2 MPa (run FL250), 353 °C at 36.7 MPa (FL350), 400 °C at 37.8 MPa (FL400), and 450 °C at 35.8 MPa (FL450). Changes in the P–T conditions were recorded in the data logger at time intervals of 0.1 s. After flashing, the autoclave was cooled to room temperature within 30 min, and the silica samples the on the alumina filter were collected from the autoclave. Observations of the surface morphology of the silica particles, and measurements of particle diameters, were carried out using a field emission–scanning electron microscope (FE–SEM, Hitachi SU-8000) at Tohoku University. The internal structures of silica particles in the flash experiment at 400 °C (run FL400) were assessed by scanning transmission electron microscope (STEM; Hitachi HD-2700A) at Tohoku University. The thin sample for STEM observation (~100 nm thickness) was prepared using a focused ion-beam instrument (FIB, Hitachi FB2000A) at Tohoku University.

### Batch experiments for silica transformation

Two series of batch experiments were conducted at 350 °C and vapour-saturation pressure (run BT350), and 450 °C (run BT450; Fig. 1; Table S1), using the products of flash experiments conducted at ~350 °C and ~450 °C, respectively. Starting silica materials for these experiments were prepared in 8–12 flash experiments at each temperature. The layers of silica particles were removed from the surface of the alumina filters to reduce the influence of alumina on silica transformation during the batch experiments. About 5 mg of silica was enclosed in the gold inner tube (2 mm diameter and 10 mm height) with holes. The inner tube and the input solution were then enclosed in a cylindrical stainless-steel autoclave with an inner volume of 8 mL (inner diameter 10.8 mm, height 100 mm; Fig. S1). The inner tube facilitated collection of the silica sample after the batch experiments. Pressure was controlled by the water-filling ratio, with pressures of run BT350 being set at the saturated vapour pressure with a water-filling ratio of 40%. Pressures in runs at 450 °C were set at the extension of the vapour-saturation curve under supercritical conditions, with a water-filling ratio of 20% (36 MPa; Fig. 1). The amount of input solutions was ~3.3 g for run BT350 and ~1.7 g for BT450, corresponding to water–rock mass ratios of ~670 and ~340, respectively. In the runs under vapour-saturated conditions, the silica sample was completely immersed in liquid. Different autoclaves were used for runs with different reaction times in individual series of batch experiments. The durations of the experiments were 1, 3, 5, 10, 15 days (Table S1). For comparison, runs were also conducted under vapour conditions at 350 °C and 5 MPa for 21 and 28 days (run BT350vap). In these experiments, the silica particles on alumina filters produced by the flash experiments were directly used as the starting silica materials, because the effect of alumina-solution interaction could be negligible under vapour condition.

After the individual runs, the surface morphology of the products was observed by field-emission scanning electron microscopy (FE–SEM; Hitachi SU-8000) at Tohoku University. Due to the rough surfaces of the products, the semi-quantitative analyses of chemical compositions were conducted by EDXS. Silica minerals were identified by micro-Raman spectrometry (Horoba XploRA PLUS) at Tohoku University, with a 532 nm laser and a 2400 grooves/mm grating. pH was measured at room temperature before and after the batch experiments.

## Supplementary information

Supplementary Information

## Data Availability

The data that support the findings of this study are available from the corresponding author upon request.
