# Supplementary material for: Silica nanoparticles produced by explosive flash vaporization during earthquakes
Source: Sci Rep. 2019 Jul 5;9:9738. doi: 10.1038/s41598-019-46320-7 (PMC6611766; doi:10.1038/s41598-019-46320-7)
Supplement: Supplementary file 1 — Supplementary Information [file 41598_2019_46320_MOESM1_ESM.pdf]

Supplementary information in *Scientific Reports*

Silica nanoparticles produced by explosive flash vaporization during earthquakes

Takashi Amagai[1,2], Atsushi Okamoto[1*], Takamasa Niibe[1], Nobuo Hirano[1], Kenichi Motomiya and Noriyoshi Tsuchiya[1]

[1]Graduate School of Environmental Studies, Tohoku University, 6-6-20, Aramaki-Aza-Aoba, Aoba-ku, Sendai, Miyagi, 980-8579, Japan

[2]Japan Oil, Gas and Metals National Corporation, 10-1, Toranomon 2-chome, Minato-ku, Tokyo, 105-0001 Japan

Table S1. Summary of the batch experiments.

Time (day)	Products[a]	Si	Al	Na	K	Fe	pH[b]
		[mmol/kg H_2O]					
Initial solution		9.19	0.22	0.29	0.15	<0.01	6.8
**BT350 (350 ˚C, 16 MPa)**							
1	AmSil	17.86	0.12	0.32	0.08	0.02	7.2
3	opal-C	18.01	0.10	0.31	0.08	0.04	7.0
5	opal-C	17.34	0.11	0.27	0.07	0.11	6.9
10	opal-C	15.61	0.11	0.28	0.07	0.07	6.8
15	opal-C	15.51	0.07	0.26	0.07	0.05	6.9
$m_{Si,eq,Qz}$	-	11.48	-	-	-	-	
$m_{Si,eq,AS}$	-	25.20	-	-	-	-	
**BT450 (450 ˚C, 36 MPa)**							
1	Qtz	4.61	0.01	0.23	0.04	0.01	6.9
3	Qtz	4.67	<0.01	0.22	0.03	0.06	NA
5	Qtz	5.44	0.01	0.42	0.06	0.02	6.7
10	Qtz	4.93	0.01	0.22	0.03	0.07	6.8
15	Qtz	5.38	<0.01	0.27	0.04	0.03	6.9
$m_{Si,eq,Qz}$		4.04	-	-	-	-	
$m_{Si,eq,AS}$		7.58	-	-	-	-	
**BT350vap 350˚C, 5 MPa)**							
21	AmSil	NA	NA	NA	NA	NA	NA
28	AmSil	NA	NA	NA	NA	NA	NA

[a] Dominant silica minerals in the products. AmSil = amorphous silica, Qtz = quartz.

[b] pH at room temperature.

NA = not analyzed

Table S2. Semiquantitative chemical compositions (mean and standard deviation) of the products of batch experiments, as measured by SEM–EDXS. Total wt.% is normalized to 100%.

Exp	BT350			BT450				
Time	1 day	5 day	15 day	1 day	1 day	10 day	15 day	15 day
Mineral	AmSil	opal-C	opal-C	Qtz	Qz+AmSil	Qz	Qtz	Qz + ANK mineral
Morphology	spherical	rough surface	rough surface	faceted	rounded	facted	facted	platy
no.	4	1	4	1	1	2	6	2
SiO_2	97.7 (0.5)	95.2	98.8 (0.3)	100.0	95.5	100.0	99.9 (0.2)	81.0 (0.8)
Al_2O_3	0.5 (0.2)	1.6	0.4 (0.2)	n.d.	1.3	n.d.	n.d.	3.5 (0.2)
Na_2O	1.2 (0.2)	1.4	0.6 (0.1)	n.d.	2.1	n.d.	n.d.	6.9 (0.9)
K_2O	0.6 (0.2)	0.7	0.3 (0.3)	n.d.	1.1	n.d.	n.d.	7.5 (0.2)
CaO	n.d.	1.0	n.d.	n.d.	n.d.	n.d.	n.d.	n.d.

AmSil = amorphous silica, Qtz = quartz

n.d. = not determined

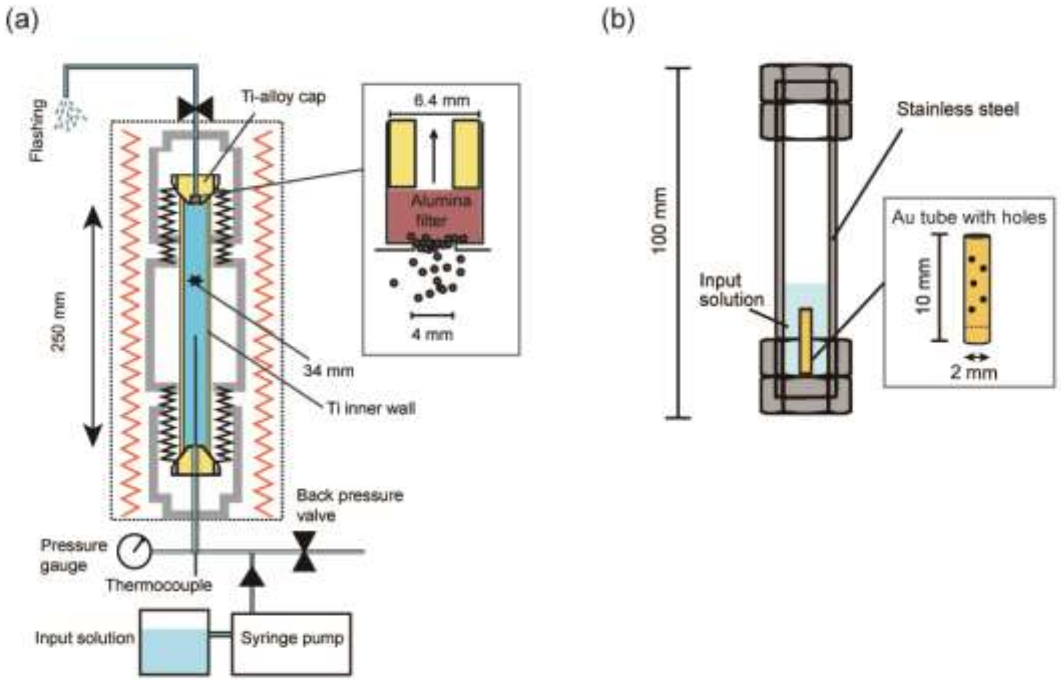

Fig. S1. Schematic illustration of the apparatus used for the (a) flashing and (b) batch experiments.

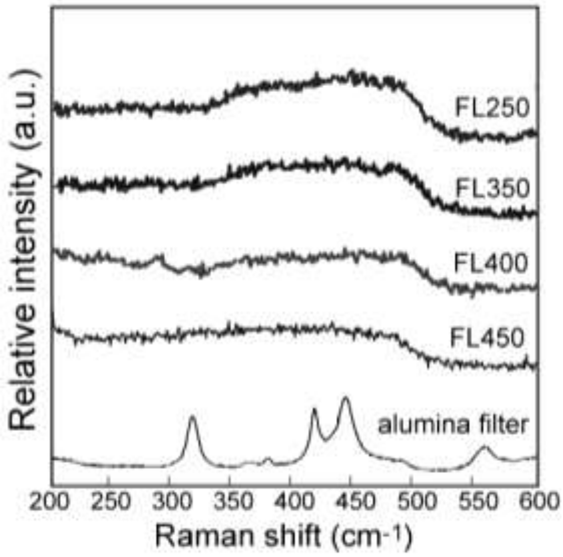

Fig. S2. Raman spectra of the products of flashing experiments (runs FL250, FL350, FL400 and FL450).
